# OLIG2 is a novel immunohistochemical marker associated with the presence of PAX3/7-FOXO1 translocation in rhabdomyosarcomas

**DOI:** 10.1186/s13000-019-0883-4

**Published:** 2019-09-07

**Authors:** Magdalena Kaleta, Anna Wakulińska, Agnieszka Karkucińska-Więckowska, Bożenna Dembowska-Bagińska, Wiesława Grajkowska, Maciej Pronicki, Maria Łastowska

**Affiliations:** 1 Department of Pathology, The Children’s Memorial Health Institute, Av. Dzieci Polskich 20, 04-730 Warsaw, Poland; 2 Clinic of Oncology, The Children’s Memorial Health Institute, Av. Dzieci Polskich 20, 04-730 Warsaw, Poland

**Keywords:** Rhabdomyosarcoma, OLIG2, ALK, TFAP2B, Immunohistochemistry

## Abstract

**Background:**

The most frequent histological types of rhabdomyosarcoma (RMS) in children are embryonal (ERMS) and alveolar (ARMS) tumours. The majority of ARMS are characterized by the presence of PAX3/7-FOXO1 gene fusion and have a worse prognosis than fusion gene-negative ARMS. However, identification of PAX3/7-FOXO1 fusion status is challenging when using formalin-fixed, paraffin-embedded (FFPE) material. Microarray analyses revealed that high expression of several genes is associated with PAX3/7-FOXO1 fusion status. Therefore, we investigated if immunohistochemical approach may detect surrogate marker genes as indicators of fusion gene-positive RMS.

**Methods:**

Forty five RMS patients were included in the analysis and immunohistochemistry was applied to FFPE tissues collected at diagnosis. Protein expression of OLIG2, a novel marker in RMS, was investigated using antibody EP112 (Cell Marque). In addition already known two markers were also analyzed: TFAP2B using rabbit anti-TFAP2β antibody (Santa Cruz Biotechnology) and ALK using anti-ALK antibody clone D5F3 #3633 (Cell Signalling). Fluorescence in situ hybridization (FISH) was performed on FFPE sections with FOXO1/PAX3 and/or FOXO1/PAX7 probes (Dual Colour Single Fusion Probe, Zytovision).

**Results:**

Our analysis revealed that all three immunohistochemical markers are associated with the presence of PAX3/7-FOXO1 fusion: TFAP2B (*p* < 0.00001), OLIG2 (*p* = 0.0001) and ALK (*p* = 0.0007). Four ARMS had negative PAX3/7-FOXO1 status and none of them displayed positive reaction with the analysed markers. Positive reaction with OLIG2 (6 tumours) was always associated with the presence of PAX3/7-FOXO1 rearrangement. Two additional OLIG2 positive cases showed inconclusive FISH results, but were positive for TFAP2B and ALK, what suggests that these tumours expressed fusion positive signature.

**Conclusion:**

Our results indicate that TFAP2B, ALK and a novel marker OLIG2 may serve as surrogate markers for PAX3/7-FOXO1 status what is especially beneficial in cases where poor quality tumour tissue is not suitable for reliable genetic analyses or shows inconclusive result.

## Background

Rhabdomyosarcoma (RMS) is the most common malignant paediatric soft tissue tumour. According to the World Health Organisation (WHO 2013) RMS includes four histopathological types: embryonal (ERMS), alveolar (ARMS), pleomorphic and spindle cell/sclerosing rhabdomyosarcoma. The first two types represent the most frequent histological categories in children, with ARMS associated with a poorer prognosis than ERMS [1, 2].

The majority of ARMS are characterized by the presence of translocations t (2;13)(q36; q14) or t (1;13)(p36;q14), which form a fusion gene between PAX3-FOXO1 or PAX7-FOXO1, respectively (previously named as PAX3-FKHR or PAX7-FKHR) [3, 4]. Remarkably, microarray-based gene expression profiling showed that fusion gene-negative ARMS (at least 20% of tumours) clustered separately from fusion gene-positive ARMS. These tumours resembled at the molecular level ERMS and patients had better prognosis than patients with fusion gene-positive ARMS [5–7]. Therefore, PAX3/7-FOXO1 status has prognostic significance in patients within the ARMS group [8–10].

Despite similar histological appearance, ARMS consist of two groups with different molecular and clinical characteristics and consequently, it is important that tumours with the presence of translocation are identified correctly. PAX3/7-FOXO1 fusion may be detected by real-time RT-PCR where frozen tumour tissue is available, but this approach is challenging when using formalin-fixed, paraffin-embedded (FFPE) material. Fluorescence in situ hybridization (FISH) on the other hand is applied on preparations from FFPE tumours, but in some cases, interpretation of the results is difficult due to poor quality of material.

Since fusion gene-positive ARMS cluster together and display distinctive gene expression signature, it is feasible to identify genes which may serve as a potential surrogate immunohistochemical markers for fusion gene-positive ARMS. Indeed, protein expression of TFAP2Β has been shown in fusion gene-positive tumours [7]. In line with this, expression of TFAP2Β at the RNA level was strongly associated with fusion gene-positive ARMS in other studies: top 1 gene in analyses by Williamsom et al., 2010 [5] and top 12th gene in analysis by Wachtel et al., 2004 [6].

Also, ALK expression at the protein and RNA levels were described as being significantly associated with the presence of PAX3/7-FOXO1 translocations [11, 12].

In this study, we investigated if other genes may serve as a potential immunohistochemical marker for fusion gene-positive RMS. We explored available expression microarrays data, performed immunohistochemical analysis and identified OLIG2 as a useful diagnostic candidate.

## Methods

### Patients and tumour material

Overall, 45 patients with RMS diagnosed in The Children’s Memorial Health Institute (CMHI) in Warsaw, Poland, were included in the analysis. Tumours were retrospectively analysed and RMS tissues were retrieved from the archives of the Children’s Memorial Health Institute’s, Warsaw, Poland, under The Bioethics Committee at the Children’s Memorial Health Institute’s approved protocol (Approval No 42/KBE/2016).

Analysis was performed on formalin-fixed paraffin embedded (FFPE) tissue samples collected at diagnosis. All tumours were retrospectively reviewed according to a recent WHO 2013 criteria [13].

### Detection of candidate genes using expression microarrays data from RMS tumours

Publicly available raw data from RMS tumours deposited in the Gene Expression Omnibus (GEO) as CEL files were re-analysed using GEO2R software. Data from GSE66533 set based on Affymetrix Human Genome U133 Plus 2.0 Array were used for analysis [14]. Comparison of genes expression level in fusion-positive and fusion negative samples was performed with Benjamini & Hochberg false discovery rate.

### Detection of TFAP2B, ALK and OLIG2 expression by immunohistochemistry

Immunohistochemical reactions were performed on 4 μm thick FFPE preparations. Expression of TFAP2B protein was detected using rabbit anti-AP-2β antibody (sc-8976, dilution 1:500; Santa Cruz Biotechnology, Santa Cruz, CA). Antigen retrieval was performed at pH 6.0 for 30 min. at 99 °C. Expression of ALK protein was detected using antibody clone D5F3 #3633 (Cell Signalling, Denver, MA, USA) at dilution 1:250 and antigen retrieval was performed using EnVision FLEX HIGH pH solution (DAKO) for 30 min. at 99 °C. Expression of OLIG2 was detected using antibody EP112 (Cell Marque) at concentration 1.92 μl/ml and antigen retrieval was performed in CC1 buffer using Ventana BenchMark ULTRA IHC/ISH system (Roche).

TFAP2B and ALK scoring were as the following: negative for < 10%, intermediate for 10–50% and positive for > 50–100% of tumour cells. OLIG2 was considered positive when > 10% of tumour cells displayed positive nuclear reaction.

Whole preparations were scanned in Hamamatsu NanoZoomer 2.0 RS scanner at original magnification 40x.

### Detection of translocations by interphase fluorescence in situ hybridization (FISH)

FISH was performed on 1 μm thick FFPE sections obtained from the same blocks as used for immunohistopathologial analyses.

For detection of t (2;13) (q36; q14) or t (1;13) (p36; q14) translocations FOXO1/PAX3 and/or FOXO1/PAX7 (Dual Colour Single Fusion Probe, Zytovision) probes we applied. Analysis was performed according to the protocols of the manufacturer of the probes.

Preparations were analysed with a fluorescence microscope F-View and Cell Sense software (Olympus).

At least 100 cells were counted for pattern of green/red signals and the positive detection of fusion was considered where at least 30% of tumour cells displayed fusion signal in tumour nuclei.

### Statistical analysis

The Fisher Exact test was performed to establish associations between variables.

## Results

### Expression microarrays data analysis and markers selection

We re-analysed data from GSE66533 set which includes 33 fusion-positive and 25 fusion negative RMS cases [14]. Table 1 presents the top 10 probes representing the top 8 genes which were significantly over-expressed in fusion-positive tumours. As expected, TFAP2B was the top highly expressed gene associated with fusion-positive tumours and was chosen for further analysis. In addition, we selected OLIG2 (top 7th gene) as a well known immunohistochemical marker for other tumours but not investigated in RMS. Also ALK (the top 30th gene) was selected, as this gene has already been described in RMS.

**Table 1:** List of the top probes/genes highly expressed in PAX3/7-FOXO1 fusion positive tumours (GEO, GSE66533 set)

	ID	Gene symbol	Adjusted P.Value	P.Value	LogFC
1	1553394_a_at	TFAP2B	6.21e-24	1.14e-28	5.992
2	214451_at	TFAP2B	1.74e-22	6.37e-27	6.809
3	231916_at	NOS1	5.63e-22	3.09e-26	5.091
4	221605_s_at	PIPOX	5.66e-22	4.14e-26	5.344
5	239132_at	NOS1	8.24e-22	7.53e-26	5.789
6	1556606_at	NAV2	9.97e-22	1.09e-25	5.598
7	228170_at	OLIG1	7.57e-21	9.69e-25	5.658
8	230076_at	PITPNM3	3.03e-19	4.44e-23	4.372
9	213825_at	OLIG2	1.41e-18	2.31e-22	4.215
10	225814_at	XRN1	8.63e-18	1.58e-21	2.160

### Detection of TFAP2B, ALK and OLIG2 expression by immunohistochemistry

All three markers were analysed in 45 RMS tumours. TFAP2B was positive (> 50% of tumour cells) in 11 cases, intermediate (10–50% of tumour cells) in 3 cases and negative (< 10% of tumour cells) in 31 cases. ALK protein expression was assessed according to the same criteria and was positive in 11 cases, intermediate in 2 cases and negative in 32 cases. OLIG2 expression was positive (> 50% of tumours cells) in 7 cases, intermediate (10–50% of tumour cells) in 1 case and negative (< 10% of tumour cells) in 37 cases. The results for individual patients are presented in Table 2.

**Table 2:** List of patients with clinical, immunohistochemical and FISH data

ID	Sex	Age yrs	Histo-logy	Stage	Meta-stases	TFAP2B IHC	ALK IHC	OLIG2 IHC	FISH PAX3/7-FOXO1 fusion
1	F	2	ARMS	IV	M1	+	+	+/−	inconclusive, FOXO1 amp.
2	M	15	ARMS	IV	M1	+	+	–	PAX3-FOXO1
3	M	14	ARMS	II	M0	+	+	+	PAX3-FOXO1
4	M	3.5	ARMS	II	M0	+	+	+	PAX3-FOXO1
5	M	1.5	ARMS	II	M1	+	+	+	inconclusive
6	M	8	ARMS	IV	M1	+	+	+	PAX7-FOXO1 and amp.
7	F	3	ARMS	IV	M1	+	+	+	PAX3-FOXO1
8	M	< 1	ARMS	IV	M1	–	–	–	negative
9	M	17	ARMS	IV	M1	–	–	–	negative
10	F	10	ARMS	IV	M1	+/−	–	–	negative
11	F	< 1	ARMS	II	M1	–	+/−	–	negative
12	M	4.5	ERMS	II	M0	+	+	+	PAX3-FOXO1
13	F	7.5	ERMS	III	M0	+	+	+	PAX3-FOXO1
14	M	12	ERMS	III	M1	+	–	–	PAX3-FOXO1
15	F	1.5	ERMS^1^	na	na	+	+/−	–	negative
16	F	4	ERMS	III	M0	–	+	–	negative
17	M	16	ERMS	II	M0	–	–	–	negative
18	M	8	ERMS	IV	M1	–	–	–	negative
19	M	14	ERMS	IV	M1	–	–	–	negative
20	M	9	ERMS	III	M0	–	–	–	negative
21	M	4	ERMS	I	M0	–	–	–	negative
22	F	5	ERMS	III	M1	–	–	–	negative
23	F	< 1	ERMS	II	na	–	–	–	negative
24	M	7	ERMS	I	M0	–	–	–	negative
25	M	7	ERMS	II	M0	–	–	–	negative
26	F	5	ERMS	I	M0	–	–	–	negative
27	M	< 1	ERMS	I	M0	+/−	–	–	inconclusive
28	F	1	ERMS	IV	M1	+/−	–	–	negative
29	M	2	ERMS	III	M1	–	+	–	negative
30	M	17	ERMS	III	M0	–	–	–	nd
31	M	< 1	ERMS	III	M0	–	–	–	nd
32	F	4.5	ERMS	IV	M1	–	–	–	nd
33	M	< 1	ERMS	I	M0	–	–	–	nd
34	M	3	ERMS	III	M0	–	–	–	nd
35	M	7	ERMS	III	M0	–	–	–	nd
36	M	3	ERMS	II	M0	–	–	–	nd
37	F	6	ERMS	III	M0	–	–	–	nd
38	M	10	ERMS	III	na	–	–	–	nd
39	M	11	ERMS	I	M0	–	–	–	nd
40	M	1	ERMS	I	M0	–	–	–	nd
41	M	11	ERMS	IV	M1	–	–	–	nd
42	F	7	ERMS	IV	M1	–	–	–	nd
43	F	15	ERMS	III	M0	–	–	–	nd
44	M	3	ERMS	II	M0	–	–	–	nd
45	F	1	ERMS	na	na	–	–	–	nd

*ARMS* Alveolar rhabdomyosarcoma; *ERMS* embryonal rhabdomyosarcoma

ERMS^1^ ERMS with alveolar variant elements

*M0* No distant metastases; *M1* Distant metastases present; *IHC* immunohistochemistry; +/−intermediate reaction present in 10–50% of cells; *FISH* Fluorescence in situ hybridization; *AMP* Amplification; *nd* Not done; *na* Not available

### Correlation between TFAP2B, ALK and OLIG2 expression, histopathology and PAX3/7-FOXO1 rearrangements

All three markers showed positive correlation with ARMS histology (*p* = 0.001).

FISH analysis was performed in 29 tumours, which included 17 tumours with positive/intermediate results for immunological markers and 12 tumours negative for all three markers. PAX3-FOXO1 fusion was detected in 7 cases, PAX7-FOXO1 fusion with amplification in one case and 18 tumours were negative for both rearrangements. In one tumour only FOXO1 amplification was detected with no status of fusion established and two other tumours showed inconclusive results. Three later cases were excluded from further statistical analysis.

**Fig. 1:**
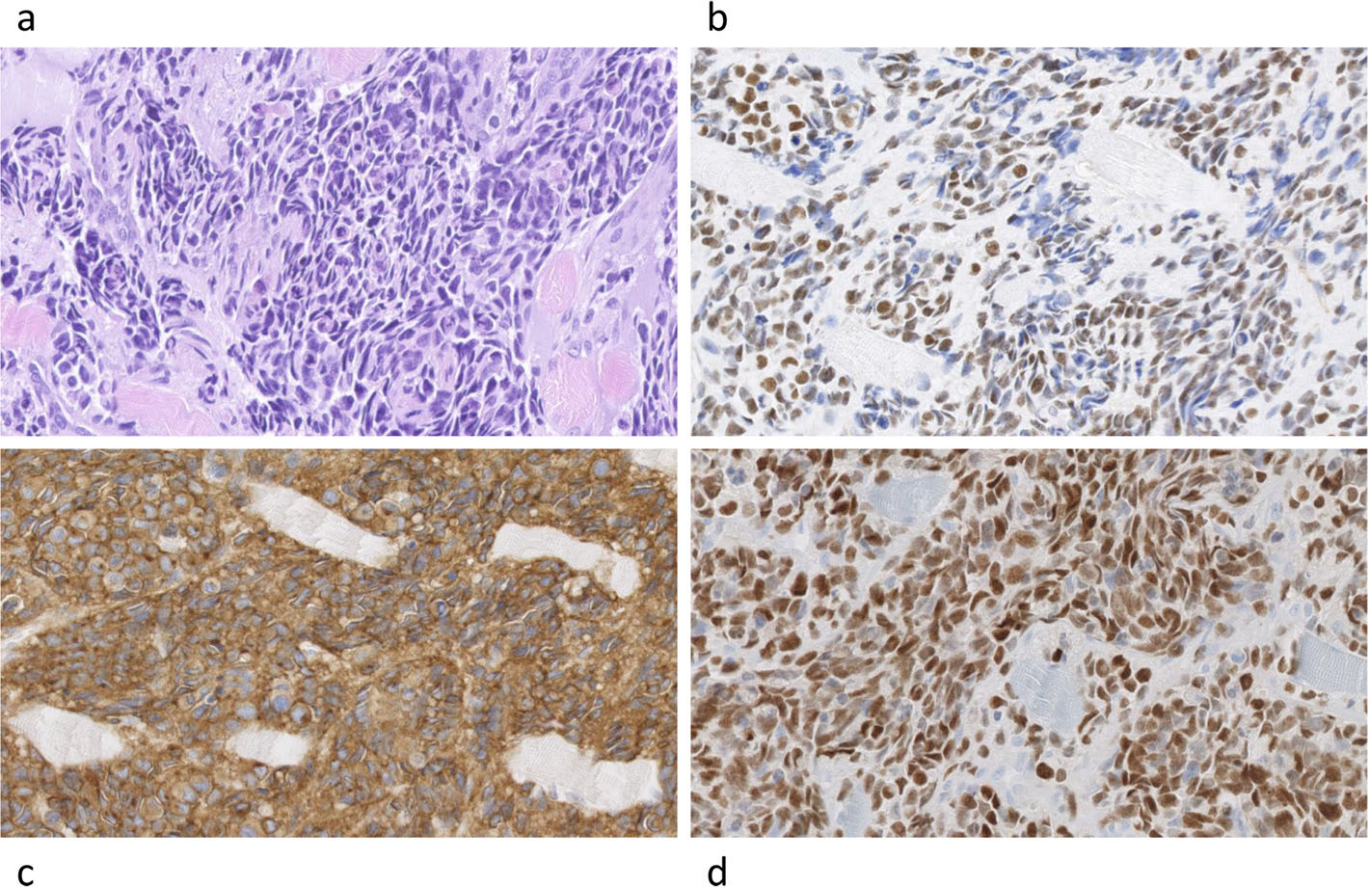
Fusion PAX3-FOXO1 positive tumour with positive immunohistopathological markers. Legend: ARMS tumour showing HE staining (**a**), positive reactions for expression of TFAP2B (**b**), ALK (**c**) and OLIG2 (**d**). Images were scanned at original magnification 40x. Digital magnification is 20x

The correlation between PAX3/7-FOXO1 rearrangements and positive reaction for immunohistochemical markers revealed significant association for TFAP2B (*p* < 0.00001), OLIG2 (*p* = 0.0001) and ALK (*p* = 0.0007), (Table 3, Fig. 1). Two tumours with solitary intermediate (10–50%) reaction for TFAP2B were fusion gene-negative. Three tumours with solitary intermediate or positive ALK reaction were also fusion gene-negative.

**Table 3:** Correlation between immunohistopathological reaction for TFAP2B, ALK and OLIG2 and PAX3/7-FOXO1 fusion status

Immunohistochemistry		Fusion positive	Fusion negative	Fisher Exact Test
TFAP2b +	9	8	1	p < 0.00001
TFAP2b -	16	0	16	
OLIG2 +	6	6	0	p = 0.0001
OLIG2 -	20	2	18	
ALK +	9	7	2	p = 0.0007
ALK -	15	1	14

We found four fusion negative cases with ARMS histopathology and none of them displayed positive reaction with TFAP2B, ALK and OLIG2. Since two patients died due to complications during treatment we can’t present survival data for this small group.

On the other hand, three fusion positive cases were detected in patients with an original ERMS diagnosis. Two cases were positive for all three immunohistochemical markers and one case was positive for TFAP2B only.

In our series one tumour displayed ambiguous histopathological morphology with a final diagnosis of ERMS with alveolar variant elements. This tumour was fusion negative, showing increased copy number (up to 10 per cell) for PAX3, PAX7 and FOXO1 and displaying TFAP2B positive reaction.

## Discussion

Detection of molecular features in RMS is of diagnostic and prognostic significance for patients. However, real-time RT-PCR approach is often not feasible since frozen tumour tissues are not routinely stored in many hospitals. Also the FFPE tumour material is sometimes of poor quality for FISH. Nevertheless, the latter tissue may still be suitable for immunohistological analysis. Indeed, in our series, we found 2 cases with the inconclusive FISH result but available immunohistological results.

Because genes expression profiles are specific for molecular subtypes of tumours, they can be explored for identification of tumours with PAX3/7-FOXO1 fusions. We confirmed the diagnostic usefulness of two already investigated immunohistological markers, TFAP2B and ALK [7, 11, 12].

TFAP2B has previously been described as a direct target gene of PAX3-FOXO1 mediating the anti-apoptotic function in RMS [15]. TFAP2B is also highly expressed in other tumours, e.g. lung adenocarcinomas and is associated with a poor prognosis for patients [16]. By contrast, the high *TFAP2B* expression is strongly associated with favourable prognosis in neuroblastoma and is linked to noradrenergic neuronal differentiation or senescence [17]. The role of this gene in different types of cancers needs further investigation.

A high ALK expression found in PAX3/7-FOXO1 fusion positive RMS collaborates with the finding of a strong PAX3-FOXO1 site in the 3rd intron of *ALK*, which is a very potent PAX3-FOXO1 dependent enhancer [18]. Therefore, in RMS both TFAP2B and ALK are functionally linked to PAX3/7-FOXO1 fusion positive tumours.

In addition to the above two genes, we found that OLIG2 may be a novel immunohistological marker for PAX3/7-FOXO1 fusion positive RMS. The gene expression microarrays studies revealed that neurogenesis-associated genes are differentially expressed when tumours are evaluated according to the PAX3/7-FOXO1 fusion status [7]. PAX3, in addition to involvement in the skeletal muscle lineage, is also involved in the development of the nervous system [19]. TFAP2B is essential for neural crest development and is expressed in the developing cerebellum. Interestingly, in the ventricular zone ~ 100% of OLIG2 expressing cells express also TFAP2B [20]. Thus, several neurogenesis genes are active in PAX3/7-FOXO1 fusion positive RMS, including OLIG2.

In our series all OLIG2 positive tumours were PAX3/7-FOXO1 fusion positive. However, in additional two OLIG2 positive cases, FISH results were inconclusive. One case showed FOXO1 amplification, but we could not detect the presence of either PAX3 or PAX7 fusion. It is likely that other rare fusion event is involved. For example, FGFR1-FOXO1 with amplification has been described in RMS [21], but this rearrangement is not routinely analyzed. Both cases were also positive for TFAP2B and ALK, what suggests that these tumours expressed fusion positive signature.

On the other hand, two cases positive for the PAX3/7-FOXO1 gene fusion were negative for OLIG2 expression. Tumour tissues were analyzed from metastases in both cases, but this may not explain OLIG2 negativity since more metastatic versus primary site cases should be investigated.

Three tumours with original ERMS diagnosis surprisingly, were positive for both PAX3-FOXO1 gene fusion and immunological markers tested. Preparations were re-examined and, on the ground of histopathological analysis alone, the diagnosis of ERMS was confirmed. Therefore, it seems that histopathological examination may be insufficient for some tumours and additional analyses presented in this paper are required for better categorization of such cases.

Our results obviously need further confirmation on the larger series of RMS tumours, nevertheless already obtained results indicate that positive OLIG2 alone or in association with other markers may serve as a substitute for the presence of PAX3/7-FOXO1 gene fusion.

## Conclusion

Our results indicate that immunohistochemical detection of OLIG2 may serve as surrogate marker for PAX3/7-FOXO1 status in RMS. This is especially beneficial in cases where poor quality tumour tissue is not suitable for reliable genetic analyses or shows inconclusive result.

## Data Availability

All data generated or analyzed during this study are included in this published article.

## Abbreviations

ARMS: Alveolar rhabdomyosarcoma
ERMS: Embryonal rhabdomyosarcoma
FFPE: Formalin-fixed, paraffin-embedded
FISH: Fluorescence in situ hybridization
GEO: Gene Expression Omnibus
RMS: Rhabdomyosarcoma
